# Photosynthetic contribution and characteristics of cucumber stems and petioles

**DOI:** 10.1186/s12870-021-03233-w

**Published:** 2021-10-06

**Authors:** Weike Sun, Ning Ma, Hongyu Huang, Jingwei Wei, Si Ma, Huan Liu, Shi Zhang, Zhenxian Zhang, Xiaolei Sui, Xin Li

**Affiliations:** 1 Department of Vegetable Science, Beijing Key Laboratory of Growth and Developmental Regulation for Protected Vegetable Crops. College of Horticulture, China Agricultural University, Yuanmingyuan Xilu 2#, HaiDian District, Beijing, 100193 China; 2 State Key Laboratory of Vegetable Germplasm Innovation, Tianjin Kernel Cucumber Research Institute, 301 Baidi Road, Nankai District, Tianjin, 300192 China

**Keywords:** Cucumber, Stem, Petiole, ^14^C, Photosynthesis

## Abstract

**Background:**

Photosynthesis in the green leafless blade tissues or organs of plants has been studied in some plants, but the photosynthetic characteristics of stems and petioles are poorly understood. *Cucurbitaceous* plants are climbing plants that have substantial stem and petiole biomass. Understanding the photosynthetic contribution of cucumber stems and petioles to their growth and the underlying molecular mechanisms are important for the regulating of growth in cucumber production.

**Results:**

In this study, the photosynthetic capacity of cucumber stems and petioles were determined by ^14^CO_2_ uptake. The total carbon fixed by the stems and petioles was approximately 4% of that fixed by one leaf blade in the cucumber seedling stage, while the proportion of the carbon accumulated in the stems and petioles that redistributed to sink organs (roots and shoot apexes) obviously increased under leafless conditions. The photosynthetic properties of cucumber stems and petioles were studied using a combination of electron microscopy and isotope tracers to compare these properties of stems and petioles with those of leaf blade using two genotypes of cucumber (dark green and light green). Compared with those of the leaf blades, the chlorophyll contents of the cucumber stems and petioles were lower, and the stems and petioles had lower chloroplast numbers and lower stoma numbers but higher thylakoid grana lamella numbers and larger stoma sizes. The Chl a/b ratios were also decreased in the petioles and stems compared with those in the leaf blades. The total photosynthetic rates of the stems and petioles were equivalent to 6 ~ 8% of that of one leaf blade, but the respiration rates were similar in all the three organs, with an almost net 0 photosynthetic rate in the stems and petioles. Transcriptome analysis showed that compared with the leaf blades, the stems and petioles has significantly different gene expression levels in photosynthesis, porphyrin and chlorophyll metabolism; photosynthetic antenna proteins; and carbon fixation. PEPC enzyme activities were higher in the stems and petioles than in the leaf blades, suggesting that the photosynthetic and respiratory mechanisms in stems and petioles are different from those in leaf blade, and these results are consistent with the gene expression data.

**Conclusions:**

In this study, we confirmed the photosynthetic contribution to the growth of cucumber stems and petioles, and showed their similar photosynthetic patterns in the terms of anatomy, molecular biology and physiology, which were different from those of cucumber leaf blades.

**Supplementary Information:**

The online version contains supplementary material available at 10.1186/s12870-021-03233-w.

## Background

Photosynthesis is the most basic physiological and biochemical metabolic pathway of plants and is of great significance for plant growth and development. The main organ of photosynthesis in plants is the leaf blade, but a large number of studies have shown that leafless green tissues or organs such as petioles, stems and green fruits also have the basic anatomical structure and physiological characteristics of photosynthesis, and provide a certain contribution to yield formation [1]; examples of these tissues, organs, and fruits include celery petioles and tobacco stems [2], cucumber fruits and immature tomato fruits [3–5], cotton bolls [6], and even fruit subsidiary tissues, including wheat awn [7] and corn husk [8]. When corn bracts were removed 15 days after flowering, the yield decreased by 17.7% [9]. The sepal is the main photosynthetic organ of hellebore (*Helleborus viridis* L.), and the contribution rate of photosynthates to the whole plant is more than 60% [10]. Some leafless tissues and organs not only play an important role in plant growth and yield formation, but also in abiotic stress conditions. In dry and hot ecosystems worldwide, the growth of many woody plants relies on stem photosynthesis [11, 12]. Some plants, such as *Brachychiton* (Malvaceae), during the dry part of the year to adapt to water stress, and the trunk photosynthesis may support the carbon needs of this plant when it does not have leaf blades [13, 14]. This scenario suggests that photosynthetic stems confer some physiological advantages, such as extra carbon fixation capacity, improved water-use efficiency (WUE) during periods when many plants are leafless, and balanced respiratory costs due to reassimilation of CO_2_ [1, 15–22]. Cucumber is a climbing plant with substantial stem and petiole biomass of stems. Old cucumber leaves become sink organs and are susceptible to diseases and insect pests. Additionally, leaf blades are delicate compared to cucumber stems and petioles. Eliminating old cucumber leaves is a regular management strategy used to prevent cucumber plants from becoming diseased, and this process can improve cucumber yields during middle and late harvest stages. Therefore, it is necessary to pay attention to the photosynthetic capacity of the leafless, green organs (stems and petioles) of cucumbers both under normal conditions and conditions when there are no leaf blades, and this capacity is an important supplement to the production capacity of leaf photosynthetic [1].

The main questions that we address in this paper are as follows: do cucumber stems and petioles have similar photosynthetic structures and functions compares to those of leaf blades? What is the photosynthetic contribution rate of cucumber stems and petioles compared to that of leaf blades, and how many of these structures contribute to sink organs? What is the difference in photosynthetic gene expression among cucumber stems, petioles and leaf blades? We hypothesized that the photosynthesis is similar in stems and petioles but different in leaf blades, and the carbon distribution shifts when plants are leafless. In the future, we expect that cucumber stems and petioles can be adjusted in terms of carbon acquisition and functional traits to be similar to those of leaf blades, but stems and petioles will maintain their physiological advantages, such as stress resistance, over leaf blades.

## Results

### ^14^C feeding in cucumber stems and petioles

To determine the presence of photosynthesis in cucumber stems and petioles, and its allocation of assimilation, under light conditions, ^14^CO_2_ was fed to different organs of a cucumber at the seedling stage (Additional file 1: Fig. S1). The different parts of cucumber irradiance were read at 48 h after a 1 h ^14^C labelling period. The stems and petioles were shown to have a positive photosynthetic capacity, although this capacity was at a low level; the assimilation of total ^14^CO_2_ by the stems and petioles was equal to 5.0 and 3.4%, respectively, of that for one leaf blade in one seedling stage in a cucumber plant, and the total of assimilation of the leafless organs was 7.6%, which was almost the sum of that of the petioles and stems (Table 1). Over half of the assimilated ^14^CO_2_ (61.6 and 55.3%) remained in the fed stems and petioles after 48 h, respectively; however, the percentage of ^14^C remaining in the leaf blade was only 14.4%. These results showed that stems and petioles were both important source and sink organs under conditions of leafless blades, and stems and petioles were the most important parts for maintaining growth after cutting off leaf blades.

**Table 1:** Distribution of ^14^C photosynthates translocated in different cucumber parts

Treatment	Shoot apex	Petiole	Stem	Root	Leaf blade	Ratio to one leaf blade
Stem & Petiole (A)	0.9% ± 0.02%	33.4% ± 3.13%	59.8% ± 4.05%	5.9% ± 0.94%		7.6%
Leaf blade (B)	0.1% ± 0.02%	16.4% ± 1.29%	67.6% ± 5.79%	1.5% ± 0.13%	14.4% ± 1.23%	100.0%
Stem (C)	2.1% ± 0.13%	34.4% ± 8.26%	61.6% ± 7.09%	2.0% ± 1.03%		5.0%
Petiole (D)	2.4% ± 0.14%	55.3% ± 1.70%	39.1% ± 2.92%	3.3% ± 0.08%		3.4%

Samples with ^14^CO_2_ absorption for stems and petioles (A), ^14^CO_2_ absorption for one leaf blade (B), ^14^CO_2_ absorption for stems (petioles are wrapped in foil to inhibition of photosynthesis) (C), ^14^CO_2_ absorption for petioles (stems are wrapped in foil to inhibition of photosynthesis) (D), all treatments retain shoot apex. The data represents mean values ± SE (n = 3)

The photosynthetic organs allocated ^14^C to the shoot apex at a much lower proportion in the leaf blade (0.1%) than in the stem (2.1%) and petiole (2.4%), and the shoot apex ^14^C feeding activity was approximately 1.5 kDPM; however, carbon distribution to the roots was substantially different, with a high carbon distribution from the leaf blades (26 kDPM) and a low carbon distribution from the stems (1.6 kDPM) and petioles (1.9 kDPM) under leafless conditions. These results may have occurred because the demand for photosynthetic products in the shoot apex was saturated at high levels of carbon fixation in the leaf blades, stems and petioles, but ^14^C assimilation transport to the root was insufficient for root growth after leaf cutting. The above results showed that cucumber stems and petioles have the capacity for photosynthesis, and photosynthetic products can contribute to sink organ growth with sufficient (shoot apex) or insufficient (root) supplementation.

### Chlorophyll content and ultrastructure of cucumber stems and petioles

**Fig. 1:**
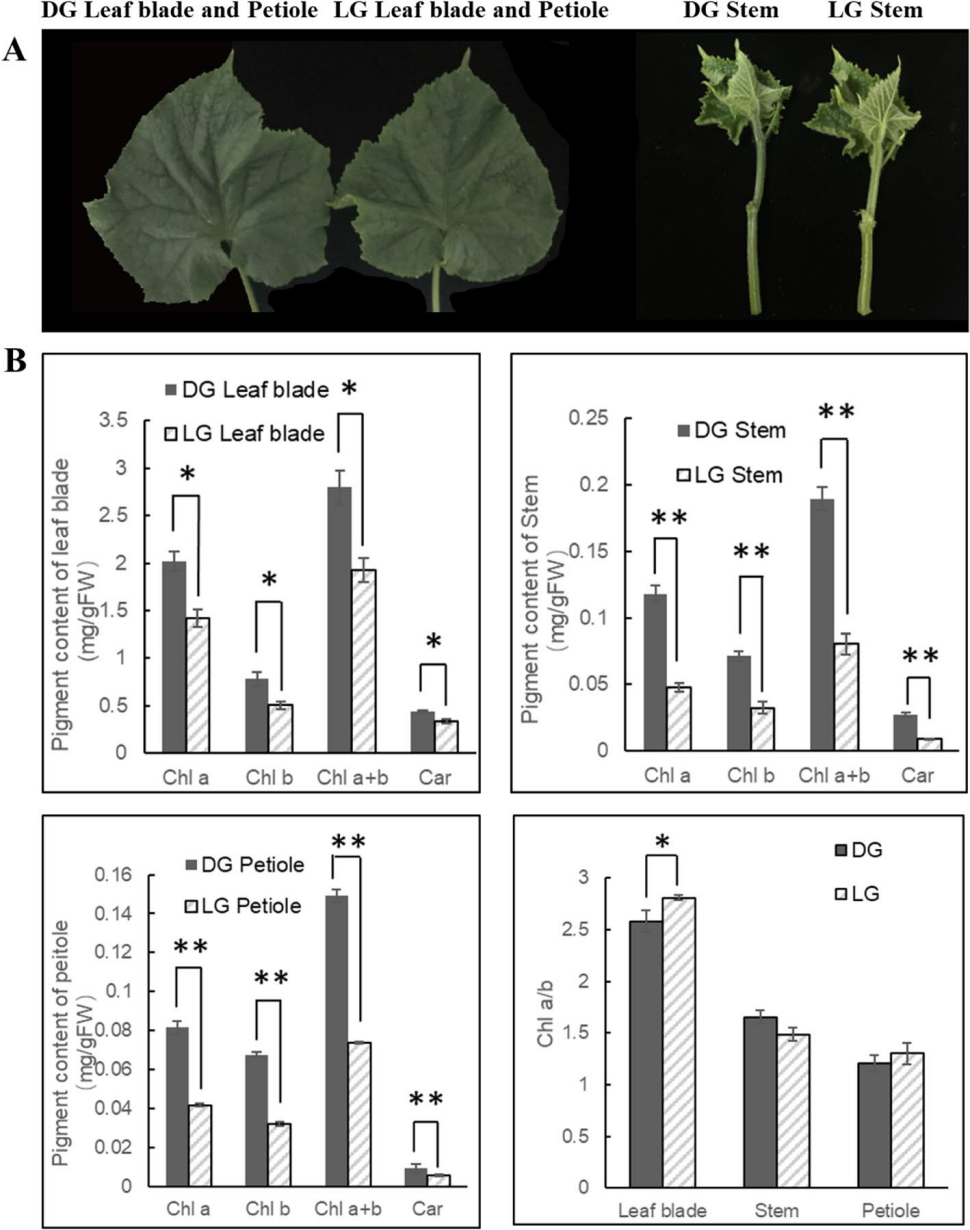
Phenotype (**A**) and pigment content analysis (**B**) in the DG and LG cucumber leaf blades, stems and petioles. Multiple comparisons were performed with significant differences (*P < 0.05, **P < 0.01, *n* = 3; according to Duncan’s multiple range test and error bars represent SE)

To explore the photosynthetic characteristics of cucumber stems and petioles, two cucumber genotypes, one dark green (DG) and one light green (LG), were used for further analysis (Fig. 1A). The chlorophyll and carotenoid contents in the DG cucumber were much higher than those in the LG cucumber (Fig. 1B) in the stems and petioles (approximately 2–3 times) and were also different in the leaf blades. The amounts of chlorophyll and carotenoids in the stems and petioles were much lower than those in the leaf blades and were approximately 4 ~ 8% (petioles or stems/leaf blades) of the weight of those in the leaf blades. Interestingly, the Chl a/b ratios were also lower in the stems (1.5–1.7) and petioles (1.2–1.3) than in the leaf blades (2.6–2.8) and showed different photosynthetic performances in these parts (Fig. 1B).

**Fig. 2:**
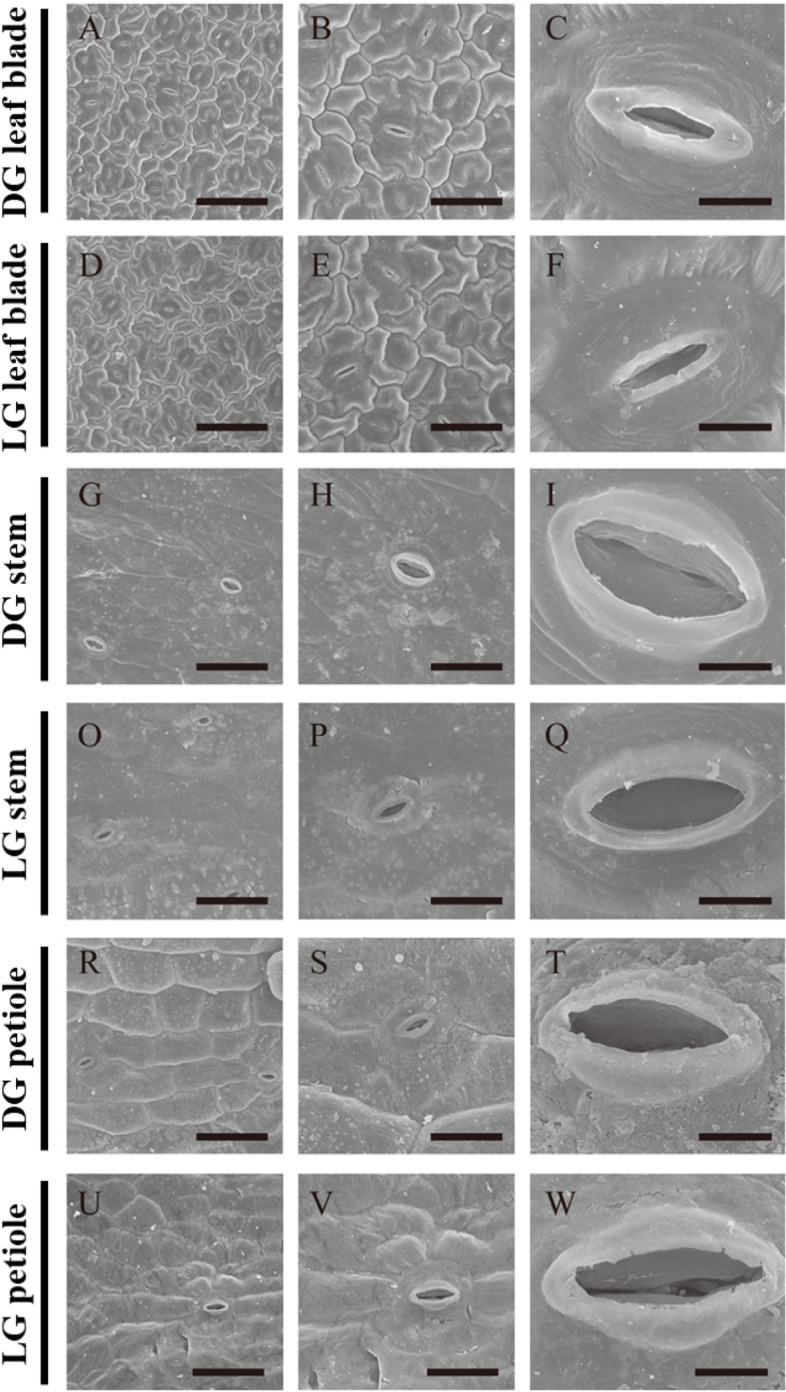
Ultrastructure of stomata in the DG and LG cucumbers. **A**, **B** and **C** the DG cucumber leaf blades; **D**, **E** and **F** the LG cucumber leaf blades; **G**, **H** and **I** the DG cucumber stems; **O**, **P** and **Q** the LG cucumber stems; **R**, **S** and **T**, DG cucumber petioles; **U**, **V** and (**W**) the LG cucumber petioles. Note: From left to right is an enlarged view, and the scale bars are 50 μm, 25 μm and 5 μm

To further explore the basic ultrastructure of the stems and petioles, we used scanning electron microscopy (SEM) and transmission electron microscopy (TEM) to observe the features of the stomata and chloroplasts in the cucumber leaf blades, stems and petioles. Stomatal frequencies on the surface of stems and petioles were only 3.1 and 1.3% of those on leaf blade, respectively (Additional file 2: Table S1), but the sizes of the stomata were about 30–90% larger on the stem (length: 10.8–12.7 μm; width 3.1–5.3 μm) and petiole (length: 10.4–12.8 μm; width 3.1–3.8 μm) surfaces than those on the leaf bade (length: 9.4 μm; width 1.2–2.4 μm) (Fig. 2, Additional file 2: Table S1). The phenomenon of large stomata is similar to that in cucumber green fruit exocarp and other species with photosynthesis in leafless organs [5, 23].

**Fig. 3:**
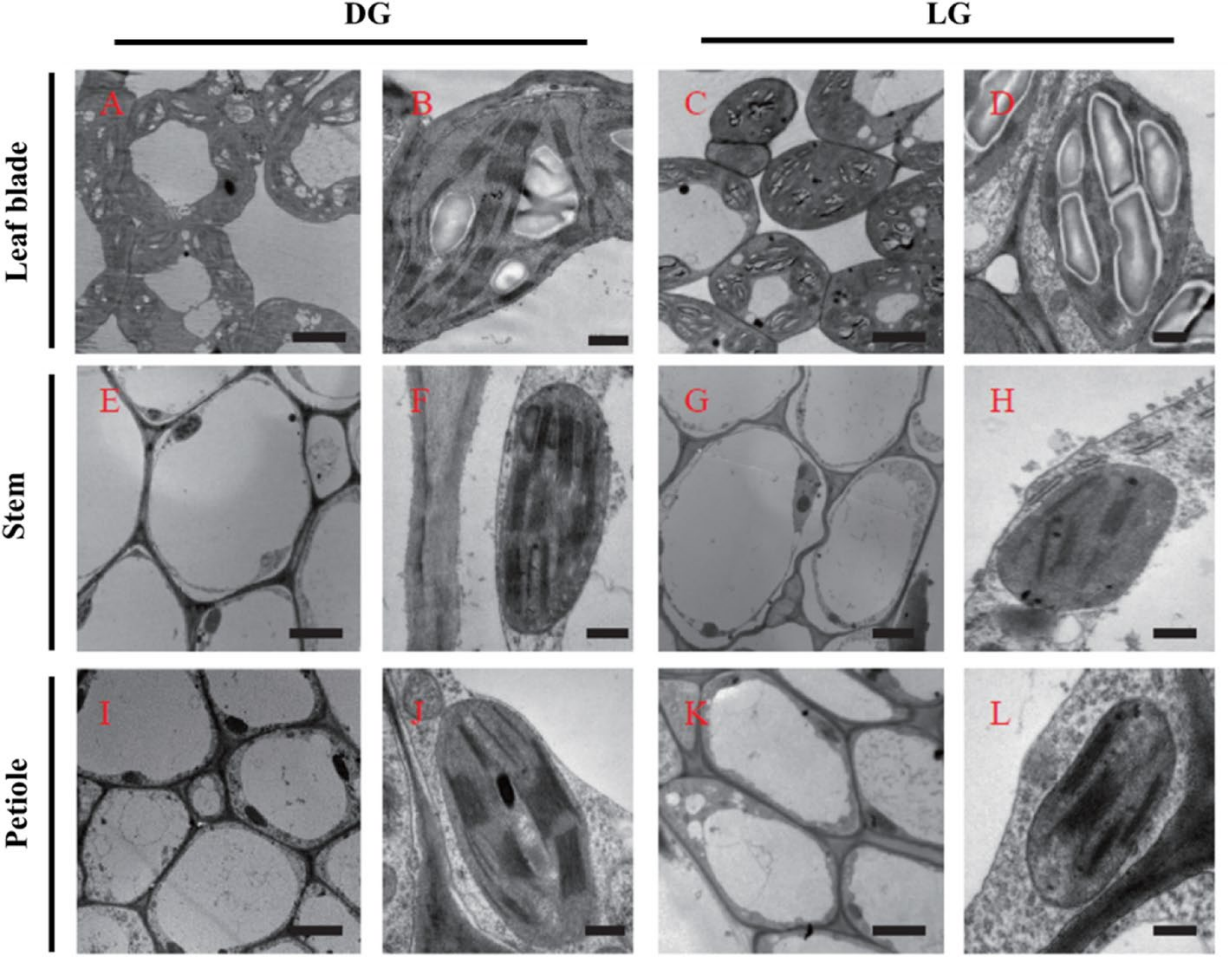
Ultrastructure of the chloroplasts in the DG and LG cucumber leaf blades, stems and petioles. Transmission electron microscopy of the DG cucumber leaf blades (**A**) and (**B**), the LG cucumber leaf blades (**C**) and (**D**), the DG cucumber stems (**E**) and (**F**), the LG cucumber stems (**G**) and (**H**), the DG cucumber petioles (**I**) and (**J**) and the LG cucumber petioles (**K**) and (**L**). Note: (**B**), (**D**), (**F**), (**H**), (**J**) and (**L**) are enlarged images of (**A**), (**C**), (**E**), (**G**), (**I**) and (**K**), respectively, and the scale bars are 5 μm and 0.5 μm for the enlarged and original images, respectively

The cells in the stems and petioles were larger than those in the leaf blades, but the chloroplast quantity per unit area in the stems and petioles was lower than that in the leaf blades (Fig. 3). In comparison to the sizes of those in the leaf blades, the sizes of the chloroplasts in the stems and petioles were smaller (Fig. 3), and the number of thylakoid grana lamellae was 1.4-and 1.7-fold larger than that in leaf blades (Additional file 2: Table S1), which is similar to the number of cucumber fruit chloroplasts and resembles that of chloroplasts in shade plants [5]. Interestingly, there was no significant difference between the DG and LG on cucumbers in terms of chloroplasts frequency, but the number of thylakoid grana lamellae was significantly lower in the different parts of the LG cucumber than in those of the DG cucumber (Fig. 3, Additional file 2: Table S1).

### Photosynthetic rate

The photosynthetic rates of the leaf blades, stems and petioles of the DG and LG cucumbers were measured by an LI-6400 portable photosynthetic measurement system. The net photosynthetic rates and respirator rates of the leaf blades were almost the same in both the DG and LG cucumbers and were calculated with a similar net photosynthetic rates of approximately 18.7 μmol m^− 2^ s^− 1^ (Table 2). Although the net photosynthetic rates in both the stems (− 0.4–0.2 μmol m^− 2^ s^− 1^) and petioles (0.2–0.4 μmol m^− 2^ s^− 1^) were at low levels, and even were negative, the total photosynthetic rate could be calculated by subtracting the respiratory rate and showed a positive photosynthetic capacity in the stems (1.7 μmol m^− 2^ s^− 1^) and petioles (1.5–1.6 μmol m^− 2^ s^− 1^). The net photosynthetic rates of the stems and petioles in the DG cucumber were significantly enhanced compared with those in the LG cucumber, but there was no significant difference in the respiration rates between these varieties (Table 2).

**Table 2:** Photosynthetic rate and respiration rate of the dark green (DG) and light green (LG) cucumber

Plant organ	Net photosynthetic rate (μmol^−2^ s^−1^)	Respiratory rate (μmol^− 2^ s^− 1^)	Total photosynthetic rate (μmol^− 2^ s^− 1^)
DG Leaf blade	18.7 ± 0.26a	−2.1 ± 0.29b	20.8
LG Leaf blade	18.6 ± 0.12a	−2.3 ± 0.03b	20.9
DG Stem	−0.2 ± 0.03c	−2.0 ± 0.14b	1.7
LG Stem	−0.4 ± 0.04d	−2.1 ± 0.08b	1.7
DG Petiole	0.4 ± 0.07b	−1.1 ± 0.13a	1.5
LG Petiole	0.2 ± 0.03bc	−1.4 ± 0.13a	1.6

The data represents mean values ± SE (n = 3) and were analysed according to Duncan’s multiple range test. Different letters indicate significant differences at *P* < 0.05

### Transcriptomic analysis of cucumber stems and petioles

**Fig. 4:**
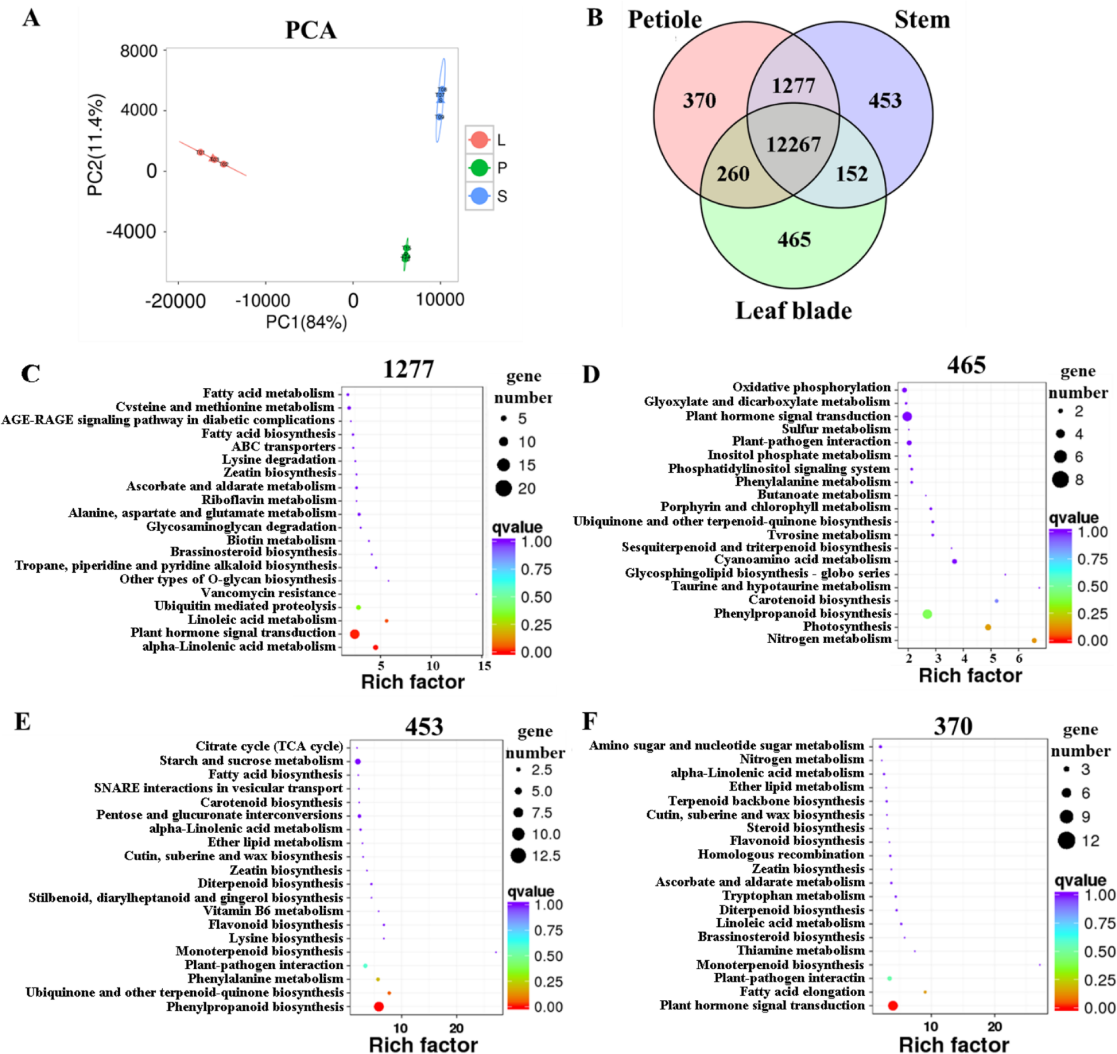
Functional comparison of coexpressed and specifically expressed genes in different organs. **A** Principal component analysis (PCA) indicates transcriptional relationships among the leaf blades (L), petioles (P) and stems (S) of a cucumber; **B** Venn diagram of the overlap genes between the DG cucumber leaf blades, stems and petioles; **C** KEGG of the coexpressed only in the DG cucumber stems and petioles (1277 genes); **D** KEGG of the genes specifically expressed in the DG cucumber leaf blades (465 genes); **E** KEGG of the genes specifically expressed in the DG cucumber stems (453 genes); **F** KEGG of the genes specifically expressed in the DG cucumber petioles (370 genes)

To further focus on the photosynthesis and its product transport in the different parts of cucumber, RNA-Seq was used to analyse the transcriptomes of the leaf blades, stems and petioles in the DG cucumber. A total of 291.8 million clean reads for each sample were generated (Additional file 2: Table S2). After removing low-quality sequences, adaptor sequences and sequence contaminants, 561.9 million cleaned reads (96.3% of the sequenced reads) were mapped to the cucumber genome. For further comparative analysis, gene expression levels were calculated using the fragments per kilobase of transcript per million fragments mapped (FPKM) reads approach. Principal component analysis (PCA) revealed that the stems, petioles and leaf blades could be classified into three separate groups (Fig. 4A, Additional file 2: Table S2), indicating that there were great differences in gene expression among the leaf blades, stems and petioles, and the samples had good repeatability.

A total of 15,244 genes were expressed in the samples, accounting for 65.6% (15,244/23248) of the annotated genes (23248) in the cucumber genome [24]. Approximately 52.8% (12,267/23248) of the annotated genes were present in all samples (FPKM≥1; Additional file 2: Table S3), and 8.4% (1277/15244) were specifically expressed in the stems and petioles. KEGG enrichment analysis showed that the specific stem and petiole expression genes were associated with responses to alpha-linolenic acid metabolism, plant hormone signal transduction, and linoleic acid metabolism (Fig. 4C).

KEGG enrichment analysis of the specific expression genes in the stems (453), petioles (370) and leaf blades (465) showed that the specific expression genes in leaf blades are most related to nitrogen metabolism, photosynthesis and carotenoid biosynthesis (Fig. 4B, D); the specific expression genes in stems are most related to phenylpropanoid biosynthesis (Fig. 4B, E); the specific expression genes in petioles are most related to plant hormone signal transduction (Fig. 4B, F). These results showed that the photosynthesis and carbon metabolism capacities in the leaf blades, stems and petioles may be different, and they are mainly carried out in the leave blades. The petioles and stems serve as supplements to the leaf blades.

**Fig. 5:**
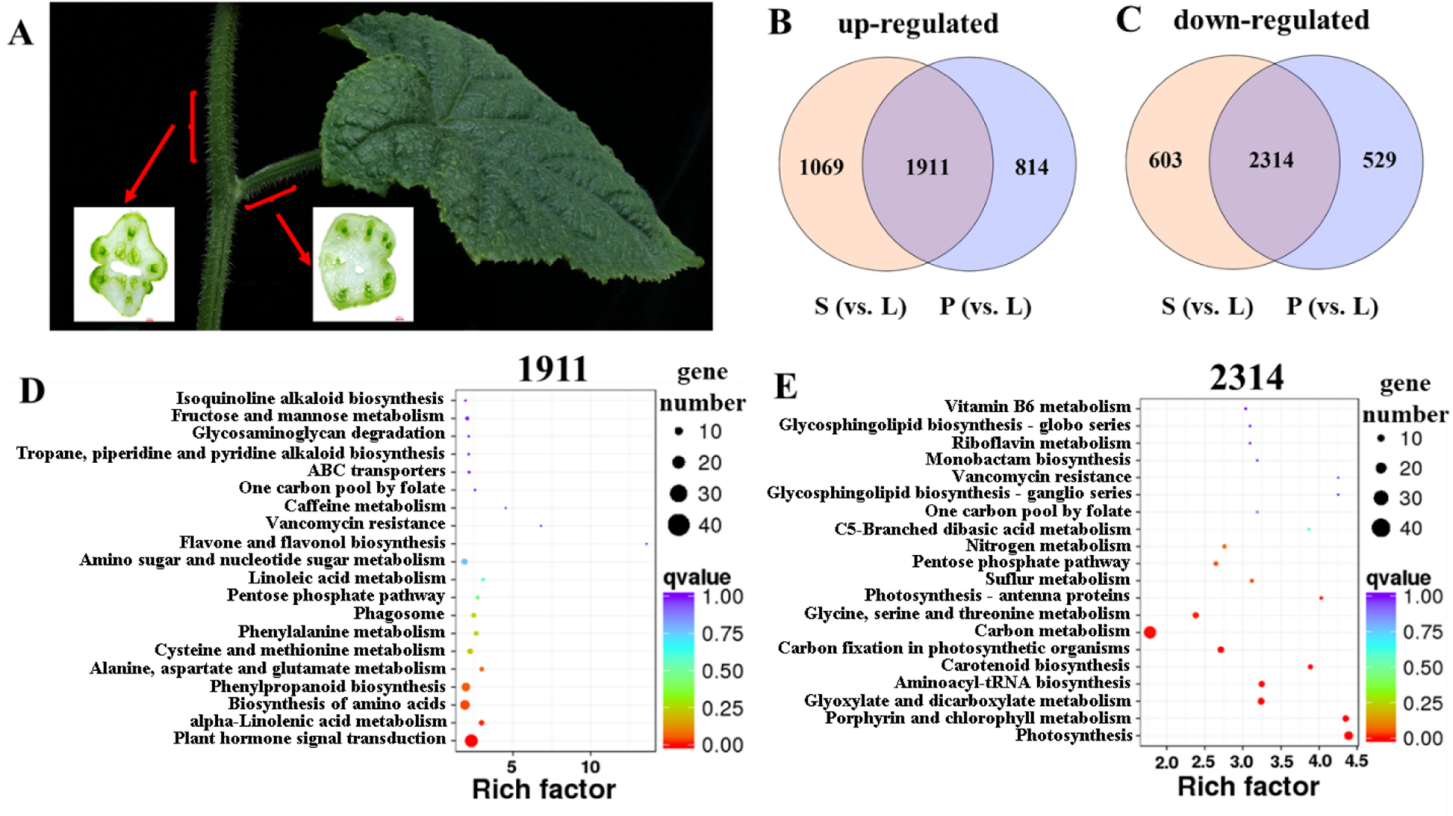
Section of stems, petioles and Venn diagram, metabolic enrichment diagram of differentially expressed (DE) genes in the DG cucumber. (**A**) left: petiole section, right: stem section. Venn diagram showing the number of genes significantly upregulated (**B**) or downregulated (**C**) in the stems and petioles compared to those in the leaf blades (L represents leaf, S represents stem and P represents petiole); KEGG diagram of the enrichment between the DG cucumber stems and petioles compared with the enrichment of the leaf blade co-upregulated genes (1911) (**D**) and co-downregulated genes (2314) (**E**)

Upregulated and downregulated genes in stems and petioles were identified (Fig. 5B, C), and 1911 (1911/23248 = 8.2%) upregulated genes were common between stems and petioles and 2314 (2314/23248 = 10.0%) downregulated genes were common between them. KEGG analysis showed that the common upregulated genes were related to plant hormone signal transduction, alpha-linolenic acid metabolism, and biosynthesis of amino acids in the stems and petioles (Fig. 5D). These upregulated genes were in similar biological pathways to those of the specific genes expressed in the stems and petioles and showed the specific characteristics of stems and petioles compared with the characteristics of leaf blades (Fig. 4 C, E, F, and Fig. 5 D). Most of the common downregulated genes between the stems and petioles were related to photosynthesis and the photosynthetic metabolic pathway, including porphyrin and chlorophyll metabolism, glyoxylate and dicarboxylate metabolism, carbon metabolism, and photosynthesis-antenna proteins, and these pathways are also in similar to those of the specific genes expressed in leaf blades (Fig. 4D, Fig. 5E).

In addition, through the cluster heatmap of upregulated and downregulated genes in the stems and petioles relative to those in the leaf blades (Additional file 1: Fig. S2), we determined that the expression of differential genes in the stems (S1–S3) and petioles (P1–P3) were obviously different from those in the leaf blades (L1–L3), which indicates that the expression patterns of differential genes in the stems and petioles are similar, but different from those in the leaf blade. At the same time, this result also shows that the three repeated samples we take had a good consistency.

### Gene expression and enzymatic activity

To further understand the regulation of gene expression in photosynthesis, respiration and chlorophyll synthesis in different green tissues of cucumber, the expression levels of some related genes were determined from RNA-Seq data.

**Fig. 6:**
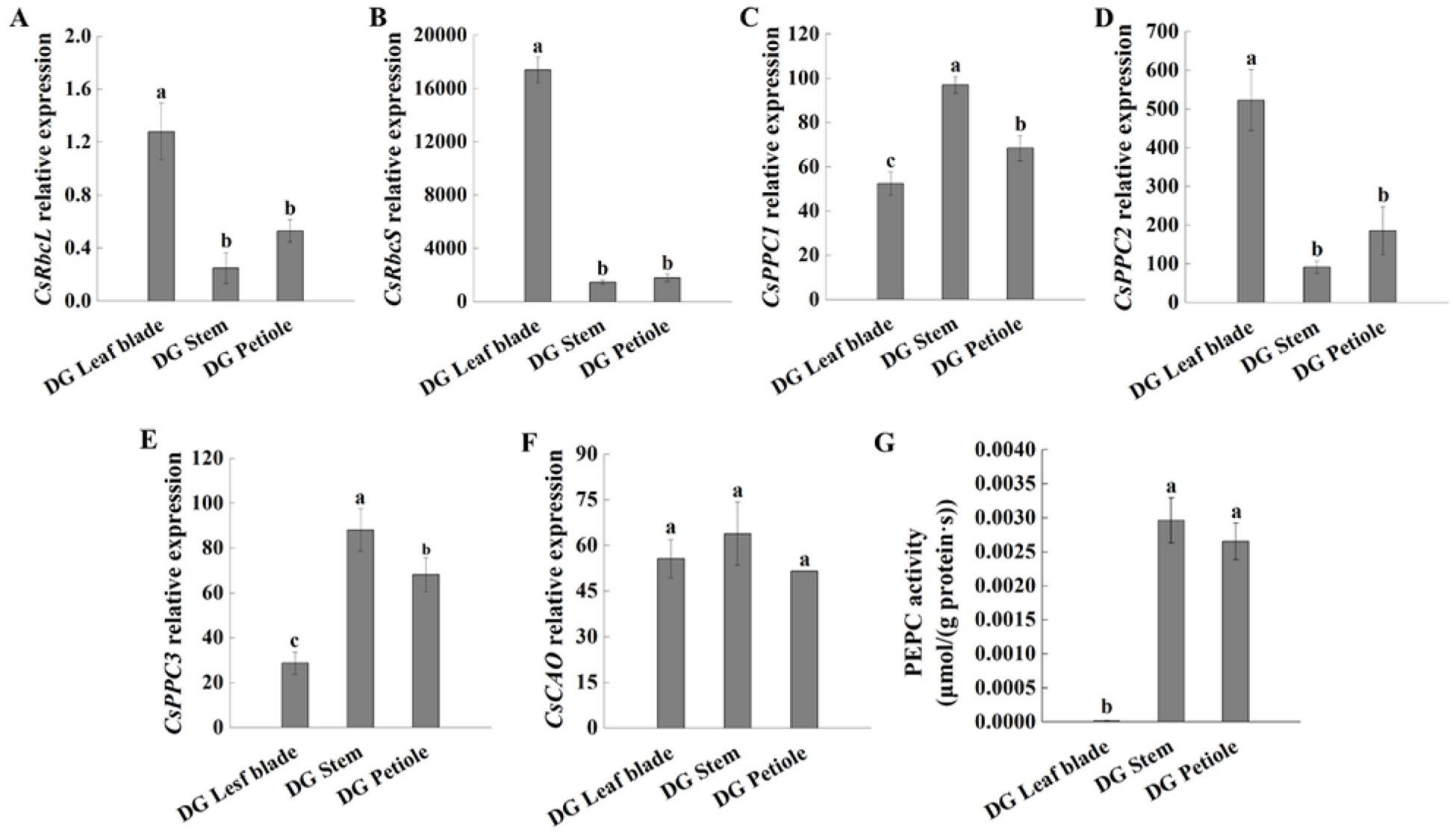
Transcriptome data of Rubisco, PEPC and *CAO* relative expression levels and enzyme activities in the dark green cucumber. Relative expression of cucumber rubisco large subunit (*CsRbcL*) (**A**), rubisco small subunit (*CsRbcS*) (**B**), *PPC1* (**C**), *PPC2* (**D**), *PPC3* (**E**) and *CAO* (**F**); enzyme activity PEPC (**G**). The data represent mean values ± SE (*n* = 3) and were analysed according to Duncan’s multiple range test. Different letters indicate significant differences at *P* < 0.05

The expression of *CsRbcL* and *CsRbcS* in the leaf blades was significantly higher than that in the stems and petioles and was associated with photosynthetic efficiency and carbon fixation capacity (Fig. 6A, B). The expression of *CsPPC1* and *CsPPC3* was higher in the cucumber stems and petioles than in the leaf blades, while *CsPPC2* expression level in the leaf blades was significantly higher than that in the stems and petioles (Fig. 6C, D, E), which is in line with the results of a previous study where *CsPPC2* was expressed mainly in leaves [5]. The enzyme activities of PEPC in the stems and petioles were higher than those in the leaf blades, which is in accordance with the strong respiration in these two parts (Fig. 6G).

CAO (chlorophyll an oxygenase) is the key enzyme involved in chlorophyll b formation from chlorophyll a. The expression level of *CsCAO* was not significantly different in the cucumber leaf blades, stems and petioles (Fig. 6F). The expression level of *CsLHCBs* was also determined, which showed a higher expression level of most *CsLHCBs* in the cucumber leaf blades than stems and petioles, except that of *CsLHCB4*, which may play an important role in petiole light harvest (Additional file 1: Fig. S3).

## Discussion

### Contribution of cucumber stems and petioles to sink organ C accumulation

The leafless green organs of many plants have basic anatomical structures and physiological characteristics for photosynthesis, which can not only contain or produce chlorophyll, but also have actual or potential photosynthetic capacity, and contribute to the carbon fixation capacity and improve plant yield [1, 25]. Cucumber petioles and stems are the main transport organ for photosynthetic products in the growth of cucumber plants, but these parts also contain chlorophyll, can undergo photosynthesis, which was confirmed by ^14^C tracer analyses in this study (Table 1), and are considered important strategies for additional C acquisition [1, 25].

To date, we had known little about how much of the leafless green organs contributed to carbon fixation and whether they were important in maintaining growth, especially under leafless conditions in climbing plants such as cucumber, which has a large proportion of stem and petiole biomass. ^14^C labeling results showed a positive carbon fixation capacity of stems and petioles, and is sufficient for shoot apex but insufficient for roots carbon demand in cucumber seedlings under leafless condition. It has been reported that stem photosynthesis contributes to bud growth on areas of defoliation, as reported for *Prunus*, *Umbellularia*, and *Arctostaphylos* species, and defoliation results in ^13^C enrichment in sugars in trunk phloem [26, 27]. Additionally, our data showed a strong carbon distribution and consumption of stems and petioles to sustain growth in all of the treatments, indicating that cucumber stems and petioles are both sink organs and source organs (Table 1). The proportion of assimilated carbon allocated to the shoot apex and roots increased under leafless conditions, indicating a change in the source-sink relationship in the stems and petioles. These results indicated that cucumber stems and petioles can not only supplement photosynthesis but also play an important role in supporting growth under leafless conditions.

### Shade-type photosynthesis in cucumber stems and petioles

The Chl a/b ratio is higher in the sun leaves than in the shade, and this ratio has been reported to be lower in the petioles (both outer chlorenchyma and inner aerenchyma) of mature winter leaves than in the lamina of *Arum italicum* [28]. Similar results were found in our data, with a lower chl a/b ratio in stems and petioles than in leaf blades. The expression level of CAO was not significantly different among the cucumber leaf blades, stems and petioles. Considering the much higher chlorophyll a content in the leaf blades than in stems and petioles, the conversion rates of Chl b from Chl a are much higher in the stems and petioles than in the leaf blades. The ultrastructure of much stronger grana stacking in cucumber stems and petioles than in cucumber leaf blades is consistent with shade-type photosynthesis, which has been reported previously [29–31]. This phenomenon has also been reported in other leafless tissues in cucumber fruits and *Arum italicum* petioles [5, 32]. If there has a same regulatory menchanism in cucumber stems and petioles needs more experiments to prove.

The LHCB antenna is involved in the regulation of light use through complex interactions in a shade-type context, and shade conditions induce an increase in the LHCB to best use the available light [33, 34]. However, the expression level of most LHCB was higher in the leaf blades than in the petioles and stems, which is consistent with a similar result in *Arum italicum* in which the LHCB protein content was 40% less in the inner aerenchyma than in the lamina [32]. Pantaleoni et al. showed that this phenomenon is accompanied by an increase in the free LHCB trimers in *Arum italicum* petioles, and the actual role of these free trimers is still being discussed [32].

Cucumbers are light-loving plants and have large size of leaf blade to absorb light. The high photosynthetic efficiency in cucumber leaf blade is consistent with its high yield and rapid fruit development. However, large leaves always cause insufficient light conditions in the lower layer of the plant canopy. Whether shade-type photosynthesis in cucumber stems and petioles is the result of long-term acclimation responses to low light is under consideration.

### The C4-like mechanisms in cucumber petioles and stems compared with C3-like mechanisms in leaf blades

The C4 pathway of photosynthesis has been reported in some leafless tissues, such as the stems and petioles of tobacco and celery [2], pericarp of barley [35], grain of wheat [36], and fruit of cucumber [5]. The ultrastructure patterns in cucumber stems and petioles (Fig. 3) are similar to those in cucumber fruits that occur at a low density with large stomata, and can minimize respiratory loss and allow photosynthetic refixation of CO_2_ before it can be released to the atmosphere [5].

Interestingly, the photosynthetic rate, chlorophyll content, chloroplast density and stomatal density of cucumber stems and petioles were all approximately 3% ~ 9% of those of leaf blades, which showed a consistent coordination between these pathways. The respiratory rates of cucumber stems and petioles also showed relationships with their diameters, which were higher in stems than in petioles.

PEPC is a key enzyme in the C4 pathway of photosynthesis and involves the initial capture of carbon to form a 4-carbon compound (oxaloacetic acid). The higher activity of the PEPC enzyme in the cucumber stems and petioles than in the leaf blades caused us to consider the C4 pathway in cucumber stems and petioles. In celery and tobacco, the vascular bundles are surrounded by Chl-rich cells that possess high activities of phosphoenolpyruvate carboxykinase (PPCK), and C4 photosynthesis in stem and petiole cells that surround the xylem and phloem of these C3 plants has been reported [2, 37]. Cucumber stems and petioles vascular bundles are also surrounded by Chl-rich cells, but with a large cavity in them (Fig. 5A). To date, we have not determined whether CO_2_ from the decarboxylation of organic acids is then refixed by Rubisco, similar to the inside of cucumber fruit, or is released into the air through the vascular organ system or the cavity in the cucumber stem and petiole.

Interestingly, most of the photosynthetic genes have similar expression patterns in stems and petioles, but these patterns differ from those in the leaf blades. Considering the similar photosynthetic structure, photosynthetic capacity, chlorophyll content and enzyme activity, the downregulated genes in the stems and petioles relative to those in the leaf blades were consistent with their chlorophyll content, photosynthetic abilities and carbon metabolism patterns.

Although a leaf blade is the main organization of photosynthesis, it is not the only organization for photosynthesis. Photosynthesis in stems and petioles serves as a supplement to that in a leaf blade. In this study, we focused on the photosynthetic contribution of stems and petioles at the cucumber seedling stage. Further study is needed at the cucumber climbing stage, which involves a large biomass of stems and petioles, and old and diseased leaf blades may be removed to adjust the source-sink relationship. Overall, the contribution of cucumber stems and petioles to photosynthesis may play more important roles in the middle and late harvest stages of cucumber than in other stages. From these data, we speculate that leaving petioles when removing old and diseased leaf blades may help supplement photosynthesis, which is the easiest way to increase individual photosynthesis and reduce disease of cucumbers and other climbing plants.

## Conclusions

This paper mainly provides relevant evidence of photosynthetic tissue anatomy, molecular biology and physiology for the photosynthesis in the leafless organs of cucumbers, i.e., stems and petioles. The contribution of stem and petiole photosynthesis to plant carbon accumulation has been discussed, and their photosynthetic complementary function under leafless conditions has been confirmed. Cucumber stem and petiole photosynthesis characteristics, including photosynthetic structure, photosynthetic capacity, chlorophyll content, gene expression and enzyme activity, have been studied and showed similar photosynthetic patterns in stems and petioles but different patterns in leaf blades, which may provide a certain theoretical basis for improving cucumber photosynthesis.

## Methods

### Plant material and growth conditions

Two cucumber genotypes, ‘dark green’ (‘DG’) and ‘light green’ (‘LG’) (cucumber seeds were acquired from Jiawang Li’s lab of Tianjin Kernel Cucumber Research Institute) were cultured in a phytotron with a 10 h photoperiod and a temperature cycle of 25/18 °C (day/night). The photon flux density (PFD) was 500 μmol m^− 2^ s^− 1^. When plants had 5–6 true leaves, the third fully expanded leaf blade, counted from the shoot tip; stems; and the corresponding petioles were collected for analysis.

For the ^14^C labelling experiment, ‘dark green’ seeds were germinated, and the cucumber seedlings were growth through hydroponics with Hoagland nutrient solution. The plants were used for the ^14^C labelling experiment when they had 5–6 true leaves.

### ^14^CO_2_ labelling

^14^CO_2_ labelling was performed as described by Zhang et al. (2004) with modifications. Four treatments were conducted to detect carbon fixation capacity in one plant for stems and petioles, one plant for leaf blades, one plant for stems only and one plant for petioles only in the cucumber seedling stage. For the stem and petiole samples, all the leaf blades were cut off, and the stems and petioles were enclosed in plastic bags (Additional file 1: Fig. S1A). For the leaf blade samples, the third cucumber leaf blades were enclosed in plastic bags, and all the other leaf blades were cut off (Additional file 1: Fig. S1B). For the stem-only and petiole-only samples, all the leaf blades were cut off, and aluminium foil was used to cover the petioles or stems to expose the corresponding stems (Additional file 1: Fig. S1C) or petioles (Additional file 1: Fig. S1D) for photosynthesis under natural light conditions. The radiolabel (1.85 MBq) was injected into a vial inside the bag, and ^14^CO_2_ was released by the addition of excess 3 M lactic acid. After the leaf blades, stems and petioles were exposed to ^14^CO_2_ for 1 h, an excess of 3 M KOH was injected into the vial to neutralize the acid and to absorb the remaining ^14^CO_2_. The bag was then removed, and the translocation of radiolabels into the roots and shoot apexs were allowed to continue for 48 h. The selected root, shoot apex, leaf blade, stem and petiole organs were sampled and rapidly frozen in liquid nitrogen, and the specific activity of ^14^C in each sample was analysed by an LS 6500 multipurpose scintillation counter. The specific activity of ^14^C was calculated in the different parts, and the ratio to a single leaf (ratio to one leaf blade = parts of cucumber/leaf blade) was also calculated. Three readings per treatment were replicated on three plants.

### Determination of pigment contents

The leaf blade, stem and petiole samples from the DG and LG cucumber varieties were obtained and immediately frozen in liquid nitrogen. The pigment contents were measured according to standard methods [38]. The pigments were extracted from small leaf pieces with 95% ethanol acetone (v/v) for at least 24 h in complete darkness at − 20 °C. The extracts were clarified by centrifugation and analysed with a Pharmacia model Ultrospec 2000 UV-Vis spectrophotometer (1 nm resolution; Amersham Biosciences, Piscataway, NJ, USA). For the Chlorophyll and carotenoid determinations, absorption was recorded at 663 nm (Chl a), 646 nm (Chl b) and 470 nm (Car), and pigment concentrations were determined with the equations reported by Lichtenthaler [38].

### Scanning electron microscopy (SEM) and transmission electron microscopy (TEM)

The surfaces of the leaf blades, stems and petioles were cut for SEM observation, and the sections of the leaf blades, stems and petioles were sampled from the DG and LG cucumber varieties for TEM. The samples were fixed with 2.5% glutaraldehyde in 0.1 M Na-K-phosphate buffer (pH 7. 2) for 2 h at 4 °C, washed with PBS (pH 7.2) three times and postfixed in 1% (v/v) OsO_4_ in the PBS (pH 7.2) at 4 °C for approximately 24 h. The samples were then dehydrated through an ethanol series (30, 50, 70, 80, 90, and 100%).

For the SEM, the samples were critical-point dried using a desiccator (HCP-2; Hitachi) and coated with gold palladium (EIKO IB-3). Stomata were observed with a HITACHI S-3400 scanning electron microscope. Stomatal frequency was calculated in 5 different fields of view per sample.

For the TEM, samples were embedded in Spurr’s resin. Ultrathin sections were cut with a LEICA UC6I microtome and viewed with a HITACHI-7500 transmission electron microscope [39]. The number of chloroplasts per unit area was calculated with 5 different microscope fields for each treatment.

### Net photosynthetic rate (Pn)

The net photosynthetic rate (Pn) was measured using an LI-6400 portable photosynthesis system (Li-Cor, Inc., Lincoln, NE, USA) equipped with an infrared gas analyser (IRGA, 6400-02B). Cucumber plants were first adapted to gas exchange light intensity conditions (usually 1 h prior to the measurement), and then, the cucumber leaf blades, petioles and stems attached to the plants were put into a chamber and sealed with plastic wrap and an additional gasket. CO_2_ concentration were maintained automatically at 400 ± 10 ppm. The measurements were taken at 28 °C. Irradiance was 1000 ± 50, μmol m^− 2^ s^− 1^, and with an air flow rate at 500 μmol s^− 1^. The gross photosynthetic rate of the sampled organs was defined as the difference between the CO_2_ evolution rates under light and dark conditions. Three readings per sample were replicated on three plants.

### Enzyme assay and immunoblotting

The enzymatic activities of the PEPC enzymes were measured as described previously [40, 41]. Frozen plant tissue was processed in ice-cold glass homogenizers with 500 μl of extraction buffer (50 mM HEPES-KOH pH 7.8, 1 mM EDTA, 0.1% Triton-X, 10 mM dithiothreitol, and polyvinylpolypyrrolidone) and 10 μl of protease inhibitor cocktail (Sigma). The homogenate was briefly centrifuged, and the supernatant was used for the assays. For PEPC, 10 μl of leaf extract was combined with 980 μl of assay buffer (50 mM EPPS-NaOH pH 8, 10 mM MgCl_2_, 0.5 mM EDTA, 0.2 mM NADH, 5 mM glucose-6-phosphate 1 mM NaHCO_3_ and 1 U ml^− 1^ malate dehydrogenase), and the reaction was initiated by the addition of 10 μl of 400 mM PEP. The activity of PEPC was calculated by monitoring the decrease in NADH absorbance at 340 nm with a spectrophotometer (Unico UV-2802PC, USA).

### RNA sequencing

The leaf blade, stem and petiole samples from the DG cucumber plants were used for RNA sequencing, each for three biological replicates. A total amount of 1 μg RNA per sample was used as input material for the RNA sample preparations. Sequencing libraries were generated using the NEBNext UltraTM RNA Library Prep Kit for Illumina (NEB, USA) following the manufacturer’s recommendations, and index codes were added to attribute sequences to each sample. Raw data (raw reads) in FASTQ format were first processed through in-house Perl scripts. The adaptor sequences and low-quality sequence reads were removed from the datasets. Raw sequences were transformed into clean reads after the data were processed. These clean reads were then aligned to the cucumber genome [42] using the Hisat2 tools software. FPKM (fragments per kilobase of exon per million fragments mapped) values of the expression genes in different organs were investigated, and FPKM values less than 1 were treated as not accumulated and discarded. For each biological replicate, the detection procedure was executed completely, and only the genes appearing in all three biological replicates were treated as final transmissible genes. Differentially expressed genes were identified with the DESeq package [43]. For further comparative analysis, GO enrichment and KEGG pathway analysis were performed using the OmicShare tools, a free online platform for data analysis (http://www.omicshare.com/tools).

## Supplementary Information

**Additional file 1: Fig. S1** ^14^C mark in the dark green cucumber. Top: plants after ^14^CO_2_ treatment, bottom: plants before ^14^CO_2_ treatment, from left to right are samples removed with the leaf blades (**A**), leaving only one leaf blade (**B**), leaving only stem (petioles are wrapped in foil to inhibition of photosynthesis) (**C**), leaving only petioles (stems are wrapped in foil to inhibition of photosynthesis) (**D**), (all treatments retain shoot apex). **Fig. S2** Differential gene expression heat map of the DG cucumber. The pigments were extracted from small leaf pieces with 95% ethanol acetone (v/v) for at least 24 h in complete darkness at − 20 °C. The extracts were clarified by centrifugation and analysed with a Pharmacia model Ultrospec 2000 UV-Vis spectrophotometer (1 nm resolution; Amersham Biosciences, Piscataway, NJ, USA). For the Chlorophyll and carotenoid determinations, absorption was recorded at 663 nm (Chl a), 646 nm (Chl b) and 470 nm (Car), and pigment concentrations were determined with the equations reported by Lichtenthaler [38]. **Fig. S3** Relative *LHCB* expression levels in transcriptome data. Relative expression of cucumber *CsLHCB1* (A), *CsLHCB2* (B), *CsLHCB3* (C), *CsLHCB4* (D), *CsLHCB5* (E), *CsLHCB6* (F) and *CsLHCB7* (G). The data represent mean values ± SE (*n* = 3) and were analysed according to Duncan’s multiple range test. Different letters indicate significant differences at *P* < 0.05

**Additional file 2: Table S1** Stoma density, chloroplast density and thylakoid grana lamellae density in different organs. The data represent mean values ± SE (*n* = 5) and were analysed according to Duncan’s multiple range test. Different letters indicate significant differences at *P* < 0.05 **Table S2** Summary of transcriptome sequencing data from dark green cucumber leaf blades, stems and petioles. **Table S3** Transcript representation in the transcriptome sequencing dataset. The average number of genes expressed in each sample, and three replicates are given in each sample.

## Data Availability

The RNA-seq data has been submitted to NCBI under the accession number of PRJNA752681 (https://dataview.ncbi.nlm.nih.gov/object/PRJNA752681).

## Abbreviations

Car: Carotenoid
Chl: Chlorophyll
*CsCAO*: Cucumber chlorophyll an oxygenase
*CsLHCB*: Cucumber light harvesting chlorophyll b
*CsRbcL*: Cucumber rubisco large subunit
*CsRbcS*: Rubisco small subunit
DEGs: Differentially expressed genes
FPKM: Fragments per kilobase of exon per million fragments mapped
Fv/Fm: Maximum potential quantum efficiency of photosystem II
FW: Fresh weight
KEGG: Kyoto Encyclopedia of Genes and Genomes
PEPC: Phosphoenolpyruvate carboxylase
*PPC*: Phosphoenolpyruvate carboxylase gene
PPFD: Photosynthetic photon flux density
PSII: Photosystem II
qP: Photochemical quenching coefficient
Rubisco: Ribulose-1,5-bisphosphate carboxylase/oxygenase
