# Supplementary material for: Photosynthetic contribution and characteristics of cucumber stems and petioles
Source: BMC Plant Biol. 2021 Oct 6;21:454. doi: 10.1186/s12870-021-03233-w (PMC8493697; doi:10.1186/s12870-021-03233-w)
Supplement: Supplementary file 1 — Additional file 1: Fig. S1 14C mark in the dark green cucumber. Top: plants after 14CO2 treatment, bottom: plants before 14CO2 treatment, from left to right are samples removed with the leaf blades (A), leaving only one leaf blade (B), leaving only stem (petioles are wrapped in foil to inhibition of photosynthesis) (C), leaving only petioles (stems are wrapped in foil to inhibition of photosynthesis) (D), (all treatments retain shoot apex). Fig. S2 Differential gene expression heat map of the DG cucumber. The pigments were extracted from small leaf pieces with 95% ethanol acetone (v/v) for at least 24 h in complete darkness at − 20 °C. The extracts were clarified by centrifugation and analysed with a Pharmacia model Ultrospec 2000 UV-Vis spectrophotometer (1 nm resolution; Amersham Biosciences, Piscataway, NJ, USA). For the Chlorophyll and carotenoid determinations, absorption was recorded at 663 nm (Chl a), 646 nm (Chl b) and 470 nm (Car), and pigment concentrations were determined with the equations reported by Lichtenthaler [38]. Fig. S3 Relative LHCB expression levels in transcriptome data. Relative expression of cucumber CsLHCB1 (A), CsLHCB2 (B), CsLHCB3 (C), CsLHCB4 (D), CsLHCB5 (E), CsLHCB6 (F) and CsLHCB7 (G). The data represent mean values ± SE (n = 3) and were analysed according to Duncan’s multiple range test. Different letters indicate significant differences at P < 0.05 [file 12870_2021_3233_MOESM1_ESM.doc]

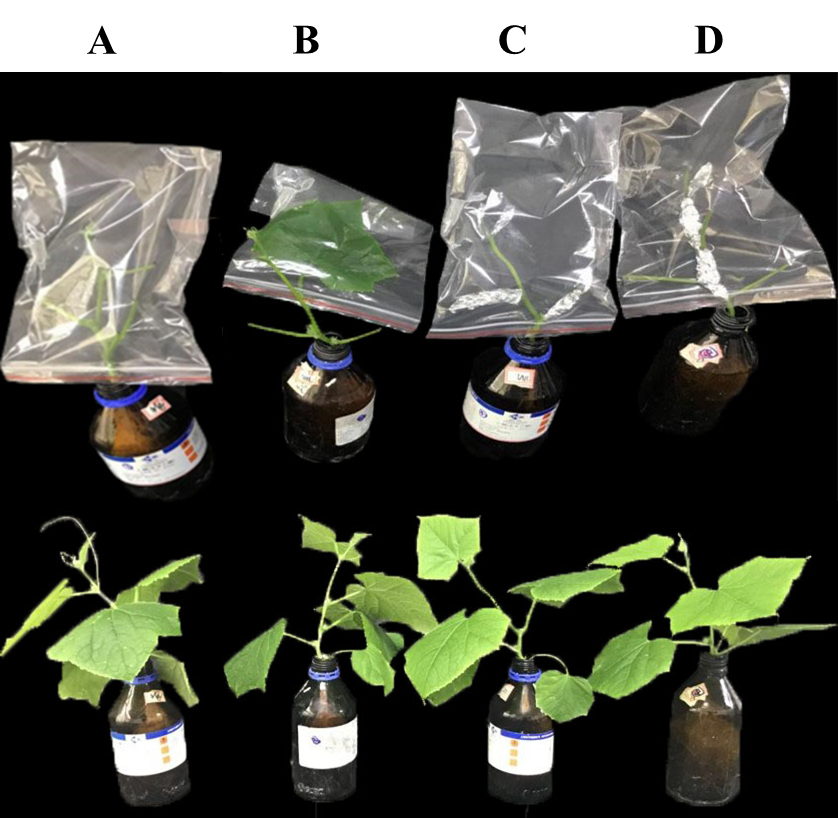


**Fig. S1** ^14^C mark in the dark green cucumber. Top: plants after ^14^CO_2_ treatment, bottom: plants before ^14^CO_2_ treatment, from left to right are samples with the leaf blades removed (A), leaving only one leaf blade (B), leaving only stem (petioles are wrapped in foil to inhibition of photosynthesis) (C), leaving only petioles (stems are wrapped in foil to inhibition of photosynthesis) (D), (all treatments retain shoot apex).


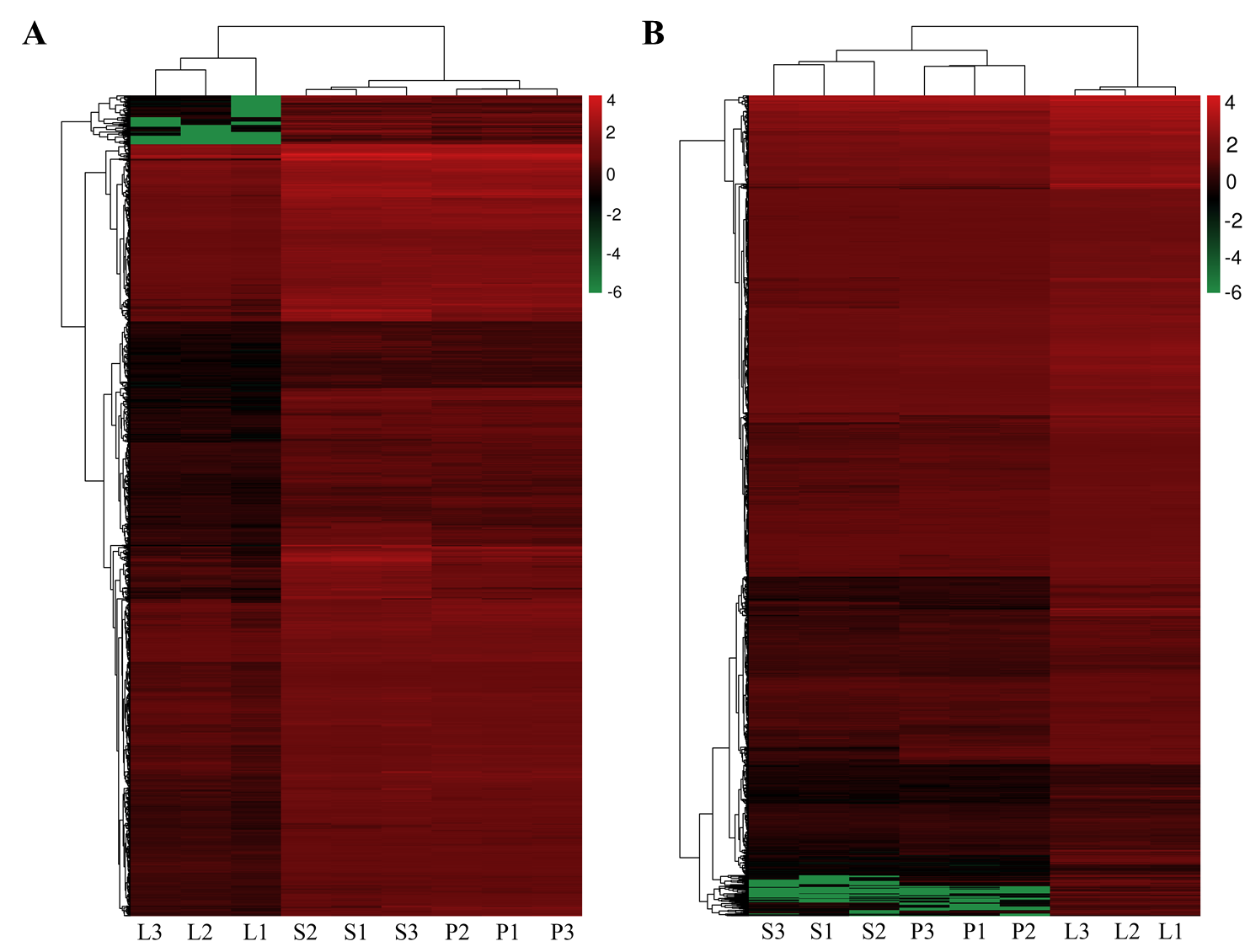


**Fig. S2** Differential gene expression heat map of the DG cucumber. Heat map showing comparisons with leaf blade, the DG cucumber stem and petiole co-upregulated genes expression (A) and co-downregulated genes expression (B) L1, L2 and L3 are the three biological duplicates of the leaf blade; S1, S2 and S3 are the three biological duplicates of the stem; P1, P2 and P3 are the three biological duplicates of the petiole.


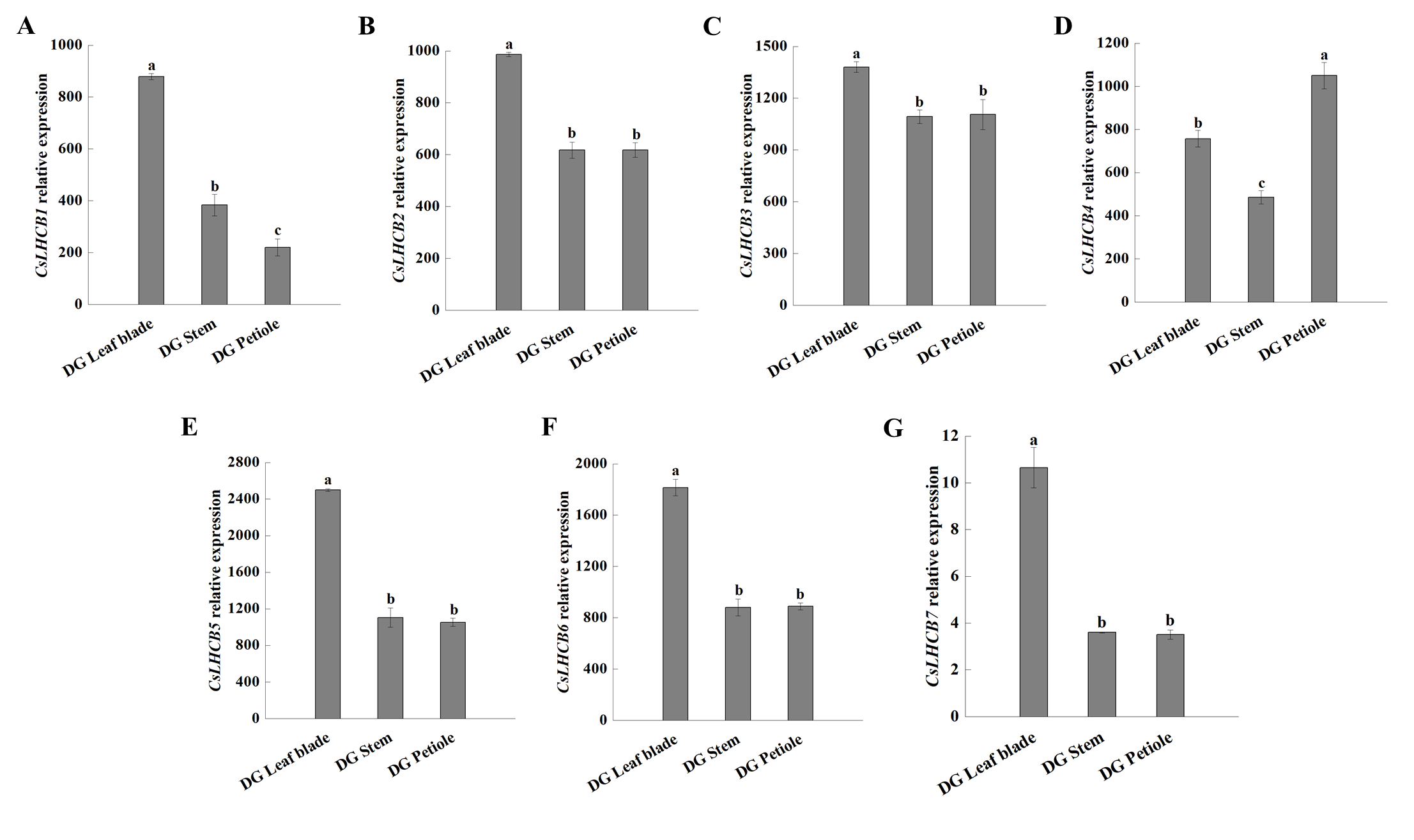


**Fig. S3** Relative *LHCB* expression levels in transcriptome data. Relative expression of cucumber *CsLHCB1* (A), *CsLHCB2* (B), *CsLHCB3* (C), *CsLHCB4* (D), *CsLHCB5* (E), *CsLHCB6* (F) and *CsLHCB7* (G). The data represent mean values ± SE (n = 3) and were analysed according to Duncan’s multiple range test. Different letters indicate significant differences at P < 0.05
