# Supplementary material for: Photosynthetic contribution and characteristics of cucumber stems and petioles
Source: BMC Plant Biol. 2021 Oct 6;21:454. doi: 10.1186/s12870-021-03233-w (PMC8493697; doi:10.1186/s12870-021-03233-w)
Supplement: Supplementary file 2 — Additional file 2: Table S1 Stoma density, chloroplast density and thylakoid grana lamellae density in different organs. The data represent mean values ± SE (n = 5) and were analysed according to Duncan’s multiple range test. Different letters indicate significant differences at P < 0.05 Table S2 Summary of transcriptome sequencing data from dark green cucumber leaf blades, stems and petioles. Table S3 Transcript representation in the transcriptome sequencing dataset. The average number of genes expressed in each sample, and three replicates are given in each sample. [file 12870_2021_3233_MOESM2_ESM.doc]

**Table S1** Stoma density, chloroplast density and thylakoid grana lamellae density in different organs.

| Plant organ | Number of stoma per unit area (1/mm^2^) | Stomatal length  (μm) | Stomatal width  (μm) | Number of chloroplast per unit area (1/mm^2^) | Number of thylakoid grana lamellae |
| --- | --- | --- | --- | --- | --- |
| DG Leaf blade | 8.3±0.09a | 9.4±0.34c | 2.4±0.49c | 295.4±31.41a | 11.3±0.67c |
| LG Leaf blade | 8.4±0.26a | 9.4±0.14c | 1.2±0.07d | 280.9±62.43a | 7.7±0.33d |
| DG Stem | 0.3±0.08b | 12.7±0.05a | 5.3±0.10a | 11.9±1.29c | 16.0±0.58b |
| LG Stem | 0.3±0.07b | 10.8±0.17b | 3.1±0.30bc | 4.0±1.29c | 8.3±0.33d |
| DG Petiole | 0.1±0.06b | 10.4±0.07b | 3.1±0.27bc | 71.3±7.95b | 19.7±0.88a |
| LG Petiole | 0.1±0.06b | 12.8±0.06a | 3.8±0.03b | 35.1±2.73bc | 12.7±0.33c |

The data represent mean values ± SE (n = 5) and were analysed according to Duncan’s multiple range test. Different letters indicate significant differences at P < 0.05

**Table S2** Summary of transcriptome sequencing data from dark green cucumber leaf blades, stems and petioles.

| Samples | Replicates | Clean reads | Mapped Reads (Mapped/Clean) | %≥Q30 |
| --- | --- | --- | --- | --- |
| DG Leaf blade | rep1 | 32,713,970 | 63,292,649 (96.7%) | 95.2% |
|  | rep2 | 29,183,569 | 56,466,531 (96.7%) | 95.1% |
|  | rep3 | 34,177,735 | 66,133,430 (96.8%) | 95.0% |
| DG Stem | rep1 | 31,867,235 | 61,497,190 (96.5%) | 95.0% |
|  | rep2 | 32,817,208 | 62,663,845 (95.5%) | 95.3% |
|  | rep3 | 32,417,544 | 62,400,796 (96.3%) | 95.0% |
| DG Petiole | rep1 | 37,250,526 | 72,018,709 (96.7%) | 94.9% |
|  | rep2 | 28,818,750 | 55,347,494 (96.0%) | 95.2% |
|  | rep3 | 32,588,998 | 62,052,858 (95.2%) | 95.0% |

**Table S3** Transcript representation in the transcriptome sequencing dataset.

| Samples | No. of Genes Represented （FPKM≥1） |
| --- | --- |
| DG Leaf blade | 13144 |
| DG Stem | 14149 |
| DG Petiole | 14174 |
| All samples | 12267 |
| At least one sample | 15244 |

The average number of genes expressed in each sample, and three replicates are given in each sample.
